# Flexible and stable PEO-based polymer composite solid electrolyte membranes incorporating NASICON-type Li_1.3_Al_0.3_Ti_1.7_(PO_4_)_3_ for high-performance all-solid-state lithium batteries

**DOI:** 10.1039/d5ra09944g

**Published:** 2026-03-05

**Authors:** Sumit Khatua, Sasikumar Karuppusamy, K. Ramakrushna Achary, Gajjala Sindhu, Tausif Alam, L. N. Patro

**Affiliations:** a Solid State Ionics Lab, Department of Physics, SRM University AP Amaravati 522240 Andhra Pradesh India patrolng@gmail.com laxminarayana.p@srmap.edu.in +91 0863-2343111 +91 0863-2343000; b SRM-Amara Raja Center for Energy Storage Devices, SRM University AP Amaravati 522240 Andhra Pradesh India; c Department of Chemistry, SRM University AP Amaravati 522240 Andhra Pradesh India

## Abstract

Research on safe, high-energy density all-solid-state lithium batteries (ASSLBs) has been rapidly advancing to address the major safety and functional limitations of conventional batteries utilizing liquid electrolytes. The development of solid electrolytes with high ionic conductivity and a wide electrochemical stability window is therefore crucial for enabling high-performance ASSLBs. In this investigation, flexible polymer composite solid electrolyte (PCSE) membranes were fabricated *via* a solution casting method using a PEO polymer matrix with optimized LiTFSI salt content, and Li_1.3_Al_0.3_Ti_1.7_(PO_4_)_3_ (LATP) ceramic filler. The PCSE membrane containing 10 wt% LATP ceramic filler (10% LATP) exhibited an ionic conductivity of 0.19 × 10^−3^ S cm^−1^ at 60 °C. The electrochemical stability of the polymer membranes was assessed *via* lithium stripping-plating experiments at 0.1 mA cm^−2^. The 10% LATP PCSE membrane demonstrated notable stability for over 900 h without significant voltage fluctuations, confirming its superior interfacial robustness with lithium. The LFP/10% LATP/Li cell at 60 °C delivered a first cycle discharge capacity of 151.6 mAh g^−1^, compared to 143.4 mAh g^−1^ for the LFP/20% LiTFSI/Li cell at 0.1C. The cell also showed remarkable rate capability and cycling performance, retaining a high capacity of 103.9 mAh g^−1^ at 1C with 86.8% capacity retention after 150 cycles. Additionally, the optimized 10% LATP PCSE membrane was also tested with a high-voltage NMC622 cathode, demonstrating its potential applicability in high-voltage ASSLBs.

## Abstract 2

Development of PEO-LiTFSI-LATP polymer composite solid electrolyte membranes for all-solid-state Li batteries.

## Graphical abstract

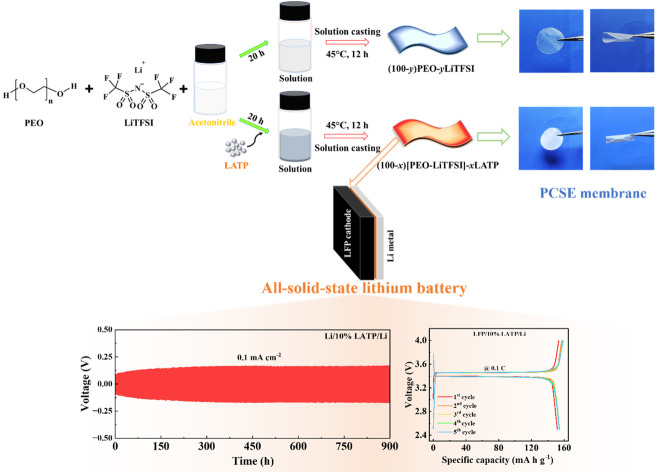

## Introduction

The demand for sustainable, high-energy-density storage devices is rapidly escalating due to their growing use in EVs and various electronic devices. Lithium-ion batteries (LIBs) have continued to dominate modern energy storage, powering diverse applications due to their high energy density, longevity, lack of memory effect, and high operating voltage.^1–4^ However, as demand surges, conventional LIBs using liquid electrolytes are nearing their energy density limits, posing significant challenges for further innovation due to their incompatibility with lithium metal anodes. Moreover, challenges such as voltage instability, lithium dendrite growth, and the formation of solid electrolyte interphase contribute to poor performance and increased safety risks. Additionally, the flammable and volatile nature of liquid electrolytes can lead to serious safety threats.^5,6^ All-solid-state Li batteries (ASSLBs), which employ solid-state electrodes and solid electrolytes, hold strong promise to revolutionize the field of energy storage by not only enhancing safety but also improving energy density.^7,8^ The major challenges in realizing ASSLBs lie in the lack of a suitable solid electrolyte that can offer high ionic conductivity and interfacial wettability with both electrodes comparable to that of liquid electrolytes.^9,10^

Solid electrolytes, the key components of ASSLBs, are broadly classified into two main types: inorganic solid electrolytes existing in both crystalline and glassy forms, and polymer electrolytes.^11^ Inorganic solid electrolytes primarily include oxides, halides, and sulphides, with oxide-based electrolytes often exhibiting notable ionic conductivity, good air stability, wide electrochemical stability windows, low interfacial resistance, and robust mechanical properties.^12^ Among various oxide-based Li-ion conducting materials, optimized systems include NASICON-type compounds such as Li_1.3_Al_0.3_Ti_1.7_(PO_4_)_3_ (LATP), and Li_1.5_Ge_0.5_Ti_1.5_(PO_4_)_3_ (LAGP), garnet-type Li_7_La_3_Zr_2_O_12_ (LLZO), and perovskite-type Li_0.33_La_0.56_TiO_3_ (LLTO), due to their good ionic conductivities and wide electrochemical stability windows.^13–15^ However, the high interfacial resistance associated with both garnet-and perovskite-based solid electrolytes, along with the use of expensive precursors (such as GeO_2_) in the preparation of LAGP, makes them less competitive than LATP. In contrast, LATP can be synthesized *via* a simple solid-state process using cost-effective precursors; moreover, it offers good ionic conductivity at room temperature, a wide electrochemical stability window, notable thermal stability, and appreciable mechanical strength.^16,17^ However, LATP is known to be unstable against lithium metal.^18^ The reduction of Ti^4+^ to Ti^3+^ has been experimentally demonstrated in numerous reports. When LATP ceramic electrolytes are directly used as solid electrolytes, interface modification is typically required, such as the use of ionic liquids or dry polymer films.^19^ LATP, like other ceramic solid electrolytes, faces several other challenges, including brittleness, the requirement for large volume in a device, and relatively high interfacial resistance with electrodes compared to polymer electrolytes, all of which hinder its commercial viability.^20^ On the other hand, polymer electrolytes offer outstanding flexibility and ensure better contact with electrodes by effectively reducing interfacial resistance, thereby enhancing overall stability of the device.^21^ They typically consist of a polymer framework incorporating a lithium salt, such as LiClO_4_, LiTFSI, or LiCF_3_SO_3_. Various polymer matrices have been demonstrated for their use in ASSLBs, including polyethylene oxide (PEO), poly(vinylidene fluoride) (PVDF), poly(vinylidene fluoride–hexafluoropropylene) (PVDF-HFP), polyacrylonitrile (PAN), polyvinylpyrrolidone (PVP), and poly(methyl methacrylate) (PMMA).^22^ Among these, polymer electrolytes based on the PEO matrix have received potential attention due to their enhanced ionic conductivity coupled with reduced interfacial resistance with electrodes, particularly when operated near their melting point (∼60 °C). Additionally, they exhibit good flexibility.^23,24^ However, the commercial potential of polymer electrolytes is largely hindered by their inadequate mechanical strength and limited ionic conductivity.^25^

Recently, extensive attention has been directed towards polymer composite solid electrolyte (PCSE) membranes, which combine an inorganic solid electrolyte (ceramic) with a polymer electrolyte to harness the strengths and benefits of both systems.^26,27^ This combination can widen the electrochemical stability window, while also improving the mechanical strength. Inorganic solid electrolytes such as LATP, which exhibit Li^+^-ion conductivity, are particularly advantageous compared to inert fillers such as alumina or silica. It not only serves as a cross-linking centre, reducing polymer crystallinity and enhancing mechanical strength, but also acts as an active electrolyte by increasing the number of charge carriers and creating pathways for rapid Li^+^-ion conduction.^28^ In addition, the reduction of Ti^4+^ to Ti^3+^ is mitigated in LATP-containing polymer membranes. The polymer wrapping effectively isolates the LATP particles from direct contact with lithium metal, thereby suppressing interfacial reduction reactions and significantly enhancing interfacial stability with lithium metal.^29^

In the current investigation, the structural and transport characteristics of PCSE membranes composed of LATP solid electrolyte, PEO polymer matrix, and LiTFSI salt were investigated using various physical and electrochemical characterization techniques. LATP was synthesized through solid-state reaction, whereas PCSE membranes were developed using a solution casting method. The optimized PCSE membrane exhibited a superior ionic conductivity of 0.19 × 10^−3^ S cm^−1^ and a wide electrochemical stability window of 4.9 V at 60 °C. Finally, the performance of the optimized PCSE membrane was evaluated by fabricating a CR-2032 type coin cell featuring a commercially procured LiFePO_4_ (LFP) cathode and a Li-metal anode, entirely free of liquid electrolytes.

## Experimental methods

### Preparation of NASICON-type LATP inorganic solid electrolyte

LATP was prepared *via* solid-state synthesis technique. Predetermined amounts of Li_2_CO_3_, Al_2_O_3_, TiO_2_, and NH_4_H_2_PO_4_ serving as precursors for Li, Al, Ti, and P, respectively, were milled at 400 rpm in a planetary ball mill for 2 h. To counterbalance for potential loss of lithium content during sintering at elevated temperatures, 10 wt% excess Li_2_CO_3_ was added. The milled powder underwent a two-step calcination process: initially at 450 °C for 2 h, followed by 850 °C for 5 h, with intermediate grinding between the steps. The calcined powder was further milled for 5 h. Subsequently, the fine powder was uniaxially pressed at 4 tons to form a round pellet with a diameter of 12 mm. Afterwards, the pellet was sintered at 1000 °C for 5 h. Finally, the sintered pellet was crushed and milled for 10 h to obtain the LATP powder for use as an active filler in the PCSE membranes. In the present study, both calcination and sintering were performed under an ambient atmosphere with heating and cooling ramp rates of 5 °C min^−1^. The pellets were not embedded in any sacrificial powder; however, a small amount of powder was placed beneath each pellet to prevent direct contact with the crucible.

### Preparation of polymer membranes

**Fig. 1:**
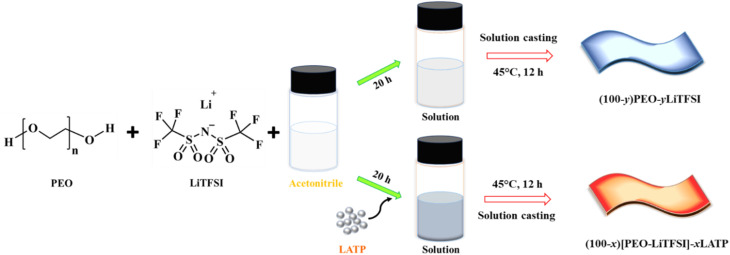
Diagrammatic representation showing the fabrication of polymer membranes based on PEO, LiTFSI, and LATP.

The polymer membranes were developed through the solution casting method, as shown in Fig. 1. Initially, the LATP powder was dispersed in acetonitrile under continuous stirring, accompanied by intermittent ultrasonication. Afterwards, LiTFSI salt and PEO polymer (MW: 600 000) were added to the dispersion, which was stirred for 20 h to achieve a homogeneous slurry. The slurry was then drop-cast onto a PTFE Petri dish and dried at 45 °C under an air atmosphere for 12 h to form flexible PCSE membranes. The membranes had an approximate thickness of 120 µm.

PEO-LiTFSI polymer membranes without LATP filler were marked as (100-*y*)PEO-*y*LiTFSI, where *y* indicates the wt% of LiTFSI salt (ranging from 0–25%). For convenience, the samples with 85% PEO-15% LiTFSI, 80% PEO-20% LiTFSI, and 75% PEO-25% LiTFSI compositions are denoted as 15% LiTFSI, 20% LiTFSI, and 25% LiTFSI, respectively. The optimized weight ratio of PEO to LiTFSI is determined to be 80 : 20 [PEO-LiTFSI]. The PCSE membranes with LATP fillers were labelled as (100-*x*)[PEO-LiTFSI]-*x*LATP, where *x* indicates the wt% of LATP (ranging from 0 to 30%). For convenience, the PCSE membranes with compositions of 90% [PEO-LiTFSI]-10% LATP, 80% [PEO-LiTFSI]-20% LATP, and 70% [PEO-LiTFSI]-30% LATP are denoted to as 10% LATP, 20% LATP, and 30% LATP, respectively. The prepared polymer membranes were stored under an argon atmosphere in glove box for further characterization and use.

### Material characterization

XRD patterns were obtained within the 2*θ* interval of 10–90° using a PANalytical Empyrean X-ray diffractometer to analyse the formation of the LATP ceramic and PEO-based polymer membranes. GSAS II software was used to perform the Rietveld refinement of LATP. Using a Bruker ALPHA II instrument, the FTIR analysis of the polymer membranes were conducted within the 400–4000 cm^−1^ wavenumber range. XPS analysis was carried out using ULVAC PHI 5000 versaprobe III instrument. Both DSC and TGA analyses were undertaken simultaneously using a NETZSCH Jupiter at a ramp rate of 5 °C min^−1^. The microstructures were visualized employing a FESEM (JEOL, JSM-7000F).

### Electrochemical characterization

Impedance measurements of the polymer membranes were conducted using a Solartron Impedance Analyzer (1260 A) from RT to 80 °C, across frequencies between 1 Hz to 10 MHz. For these tests, the polymer membranes were mounted between stainless steel (SS) blocking electrodes. The ionic conductivity (*σ*_*t*_) of the polymer membranes was evaluated *via* the relationship: 
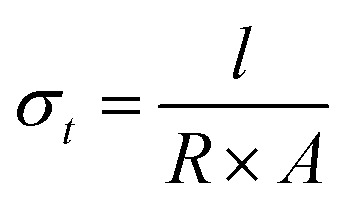
, where *A* corresponds to the cross-sectional area and *l* represents the thickness of the polymer membrane. The resistance, *R,* at a typical temperature was obtained by fitting a suitable equivalent circuit to the complex impedance isotherm (Nyquist plot). The Li^+^-ion transference number (*t*_Li_^+^) of the polymer membranes at 60 °C was measured through a Metrohm Autolab workstation using Li/Polymer membrane/Li symmetric cell configurations and calculated using the Bruce–Vincent equation:^30^1
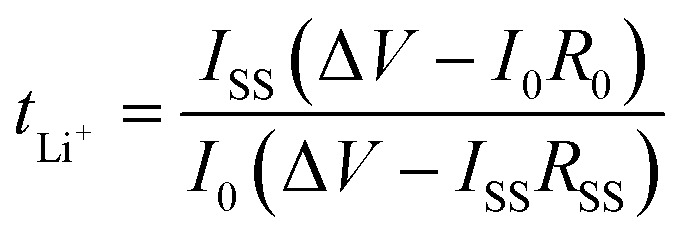
Here, *R*_0_ represents the initial resistance, and *R*_SS_ is the steady-state resistance after perturbation, both obtained from impedance spectroscopy. *I*_0_ and *I*_SS_ denote the initial and steady-state currents, respectively, obtained from *dc* polarization at a polarization voltage of 50 mV. Linear sweep voltammetry (LSV) experiments were conducted using a Metrohm Autolab at a scan rate of 0.1 mV s^−1^ to assess the electrochemical stability window of the polymer membranes over a potential window of 2–5.5 V. The LSV measurements were executed using a SS/Polymer membrane/Li cell configuration, where SS served as the working electrode and lithium metal as the counter electrode. SS/Li configurations have been employed in several reports to determine the electrochemical stability window of polymer membranes using LSV.^23,24,31^ Méry *et al.* previously reviewed the limitations of this configuration. In particular, overestimation due to the small effective surface area of the SS electrode, resulting from its flat surface, cannot be ruled out.^32^ Critical current density (CCD), rate performance, and cycling stability of the polymer membranes was obtained using Li/Polymer membrane/Li symmetric cell configurations. The suitability of the developed polymer membranes as solid electrolytes in ASSLBs was further evaluated by fabricating CR2032-type coin cells. To prepare the cathode slurry, active material (LFP or NMC622), Super P, and PVDF were mixed in a 70 : 20 : 10 weight ratio with NMP as the solvent. The slurry was then spread onto aluminium foil and oven-dried at 60 °C for 12 h. The dried electrode coating was subsequently punctured into circular discs with a diameter of 15 mm, with a mass loading of 1.5–2 mg cm^−2^ active material. ASSLBs were assembled in CR2032-type coin cells using the circularly cathode discs, polymer membranes, and lithium metal anodes, inside a controlled glove box environment, maintaining O_2_ and H_2_O levels kept under 0.1 ppm. Charge–discharge tests were conducted at 60 °C using a Neware battery testing unit over a potential window of 2.5–4.0 V. Impedance measurements of coin cells were performed using Metrohm Autolab in the frequency range of 100 KHz to 100 mHz at 60 °C.

## Results and discussion

**Fig. 2:**
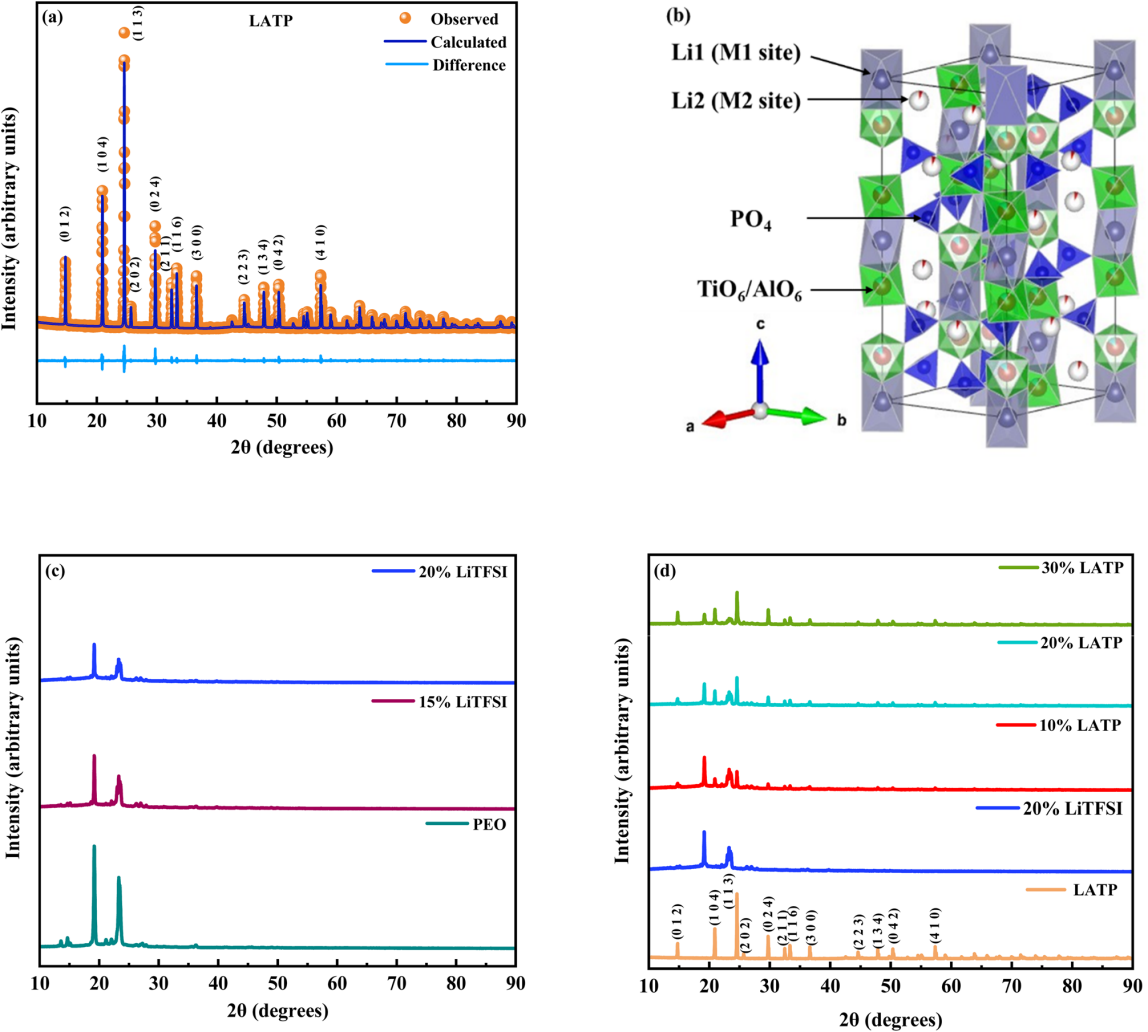
(a) Observed XRD pattern and Rietveld-refined pattern of LATP. (b) Crystal structure of LATP. (c) XRD profiles of bare PEO, 15% LiTFSI, and 20% LiTFSI polymer membranes. (d) XRD patterns of 20% LiTFSI, 10% LATP, 20% LATP, and 30% LATP polymer membranes, along with LATP ceramic.

XRD was deployed to study the phase formation and crystallinity of the LATP ceramic and the PEO-based polymer membranes. Fig. 2(a) shows the observed and Rietveld refined XRD patterns of LATP, verifying the successful formation of the phase crystallized in rhombohedral crystal symmetry with space group of *R*3̄*C*. No impurity peaks were observed. The goodness of fit was found to be 3.0, indicating the excellent matching between the observed and calculated XRD patterns. The lattice parameters of LATP were determined to be *a* = *b* = 8.4984(9) Å and *c* = 20.8119(5) Å, consistent with reported values.^33^

LATP belongs to the NASICON-family of materials, featuring a 3D framework of corner-sharing TiO_6_/AlO_6_ octahedra and PO_4_ tetrahedra (Fig. 2(b)). The LATP structure offers two sites for Li^+^ ions: the M1 site, positioned between the two TiO_6_ octahedra and coordinated by six oxygen atoms, and the M2 site, located perpendicular to the *c*-axis and coordinated by eight oxygen atoms. The migration of Li^+^ ions within the LATP structure occurs *via* the coordinated M1-M2-M1 route.^34^Fig. 2(c) and (d) show the XRD profiles of the PEO-LiTFSI, and PEO-LiTFSI-LATP polymer membranes, respectively. The intensity of the major characteristic XRD peaks for PEO-based polymer membranes at ∼19° and ∼23° decreases with increasing LiTFSI concentrations (Fig. 2(c)). This not only signifies the successful incorporation of lithium salt into the PEO polymer matrix but also indicates a suppression of crystallinity within the PEO polymer matrix upon the lithium salt addition. The XRD patterns of 10% LATP, 20% LATP, and 30% LATP PCSE membranes show the significant presence of the major characteristic peaks of both PEO and LATP (Fig. 2(d)). The characteristic peaks of LATP appear more prominent with a higher proportion of LATP in the polymer matrix.

**Fig. 3:**
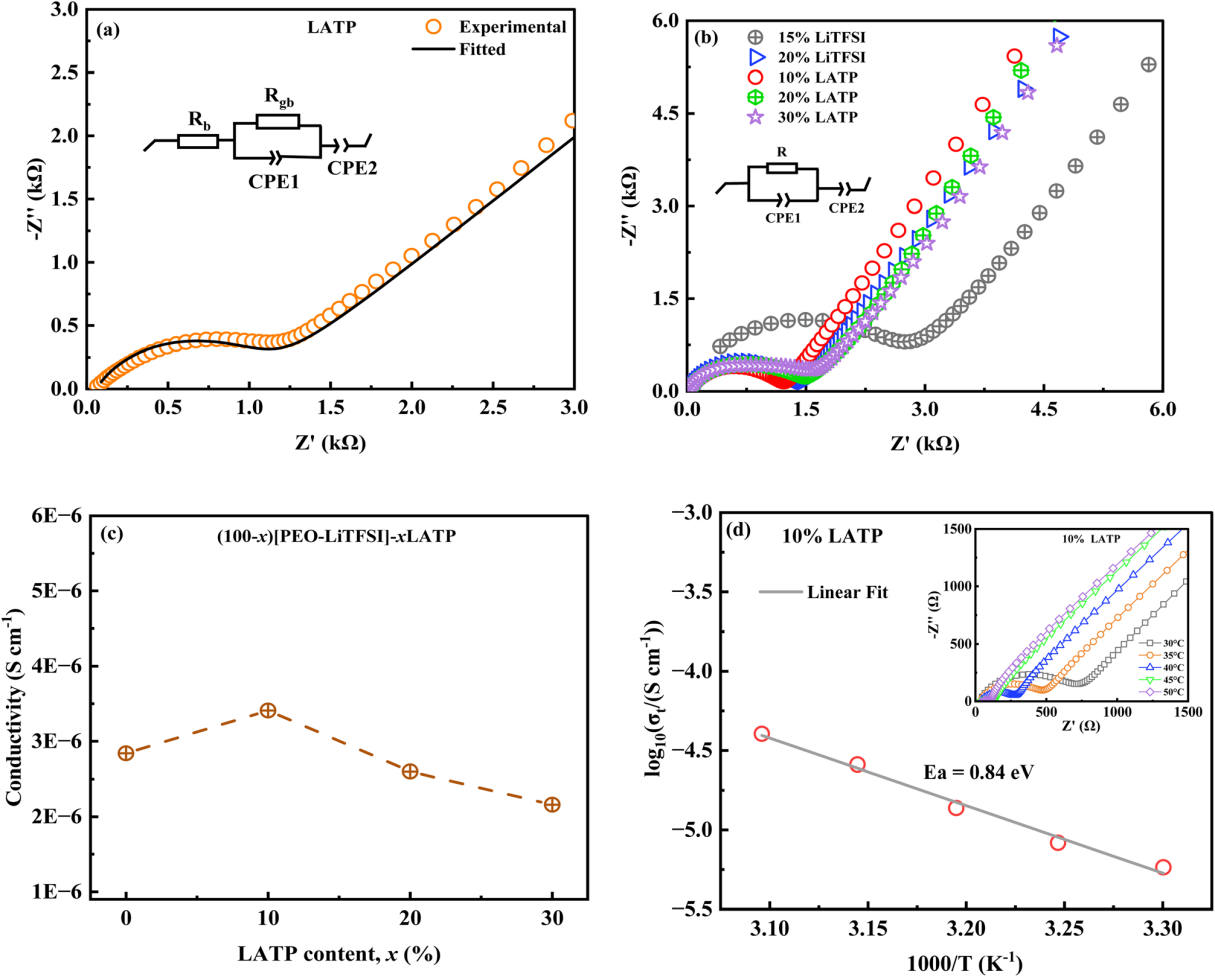
(a) Complex impedance plot of LATP at RT. (b) RT complex impedance plots of PEO-LiTFSI, and PEO-LiTFSI-LATP polymer membranes. (c) RT ionic conductivity of PEO-LiTFSI-LATP PCSE membranes at different LATP concentrations. (d) Plot of the logarithm of ionic conductivity *versus* 1000/T for 10% LATP PCSE membrane. Inset: complex impedance plots of 10% LATP PCSE membrane at different temperatures.

The complex impedance plot of the LATP inorganic solid electrolyte at RT is depicted in Fig. 3(a). The Nyquist plot displays a depressed semicircle at high frequencies and a tail-like feature at low frequencies, indicating the electrode–electrolyte interfacial response.^35^ The resistance (*R* = *R*_b_ + *R*_gb_) of LATP was determined from the low-frequency intercept of the depressed semicircle, considering the pellet's dimensions (thickness and cross-sectional area), the conductivity of the LATP pellet was calculated to be 0.72 × 10^−4^ S cm^−1^ at RT. The temperature dependent conductivity plot of the LATP pellet, exhibiting Arrhenius behaviour, is shown in Fig. S1. The activation energy was found to be 0.30 eV.

The RT complex impedance plots of the synthesized polymer membranes, shown in Fig. 3(b) exhibit a semicircular arc at high frequencies and a tail-like part at low frequencies. The equivalent circuit used for the impedance analysis consists of CPE2, which acts as a blocking double layer capacitance, in series with a parallel combination of bulk resistance (*R*) and a constant phase element (CPE1). The CPE is generally considered a leaky capacitor (*i.e.*, a hybrid between a resistor and a capacitor). The conductivities exhibited by the various polymer membranes are summarized in Table S1. The 20% LiTFSI polymer membrane exhibits higher conductivity than the 15% LiTFSI polymer membrane due to the higher LiTFSI salt concentration. In PEO-LiTFSI membranes, the LiTFSI salt dissociates into Li^+^ and TFSI^−^ during preparation, increasing the number of Li^+^ charge carriers. It also increases the amorphous content of PEO, which enhances polymer chain mobility and allows Li^+^ ions to move more easily.^36,37^ The fabrication of PEO-LiTFSI polymer membranes is limited with 20 wt% LiTFSI salt content. PEO-LiTFSI polymer membrane with 25 wt% LiTFSI salt content (25% LiTFSI) not only shrinks but also sticks to the PTFE Petri dish (Fig. S2). Consequently, the 20% LiTFSI was chosen for further development of PCSE membranes, incorporating LATP fillers of various concentrations. The RT conductivity value exhibited by the 20% LiTFSI polymer membrane without active LATP filler is found to be 0.28 × 10^−5^ S cm^−1^. Fig. 3(c) shows the variation of RT ionic conductivity of PEO-LiTFSI-LATP PCSE membranes with different LATP concentrations. Among these, the 10% LATP PCSE membrane exhibits the highest ionic conductivity of 0.34 × 10^−5^ S cm^−1^ at RT. The inset of Fig. 3(d) shows the complex impedance plots of 10% LATP PCSE membrane at various temperatures. The variation of conductivity with temperature (RT-50 °C, below the melting temperature) for 10% LATP PCSE membrane is shown in Fig. 3(d). The conductivity increases with temperature due to enhanced ion mobility and increased polymer chain segmental motion.^30^ The temperature-dependent ionic conductivity (30–80 °C) plots of PCSE membranes with varying LATP ceramic filler concentrations are shown in Fig. S3.

**Fig. 4:**
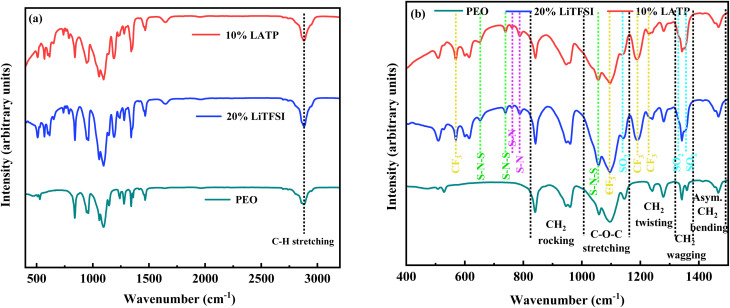
(a) FTIR spectra and (b) corresponding zoomed view of selected regions for pure PEO polymer, 20% LiTFSI, and 10% LATP polymer membranes.

FTIR spectroscopy was utilized to identify the functional groups present in the polymer membranes (Fig. 4(a)). In all cases, the peaks located around 839 and 949 cm^−1^ are corresponding to CH_2_-rocking vibrations. The peaks around 1240 and 1279 cm^−1^ are associated with CH_2_-twisting, while those near 1341 and 1359 cm^−1^ are corresponding to CH_2_-wagging vibrations. The peak around 1466 cm^−1^ is due to the asymmetric CH_2_-bending vibrations. The peak near 2873 cm^−1^ is attributed to C–H stretching vibrations of CH_2_, whereas the peaks at 1058, 1097, and 1146 cm^−1^ are assigned to C–O–C vibrations of PEO.^38–42^ Due to the presence of LiTFSI in both the 20% LiTFSI and 10% LATP polymer membranes, additional peaks appear at 570, 1092, 1190, and 1229 cm^−1^ corresponding to the CF_3_ group; 652, 738, and 1056 cm^−1^, corresponding to the S–N–S group; 762 and 787 cm^−1^ corresponding to the S–N group; and 1140, 1329, and 1354 cm^−1^, corresponding to SO_2_ group.^38,39,43^

Additionally, it is observed that the addition of LiTFSI salt leads to the changes in peak intensity and peak broadening in the PEO spectrum (Fig. S4). This behaviour arises from interactions between the salt's cations and anions and the coordinating sites of PEO.^42^ The FTIR spectra of the 20% LiTFSI and 10% LATP polymer membranes appear identical, indicating LATP is dispersed within the PEO-LiTFSI matrix. (Fig. 4(b)). The addition of LATP to the PEO-LiTFSI PCSE membrane reduced the peak intensities, suggesting a decrease in crystallinity due to disruption of the ordered chain structure (Fig. S4).

**Fig. 5:**
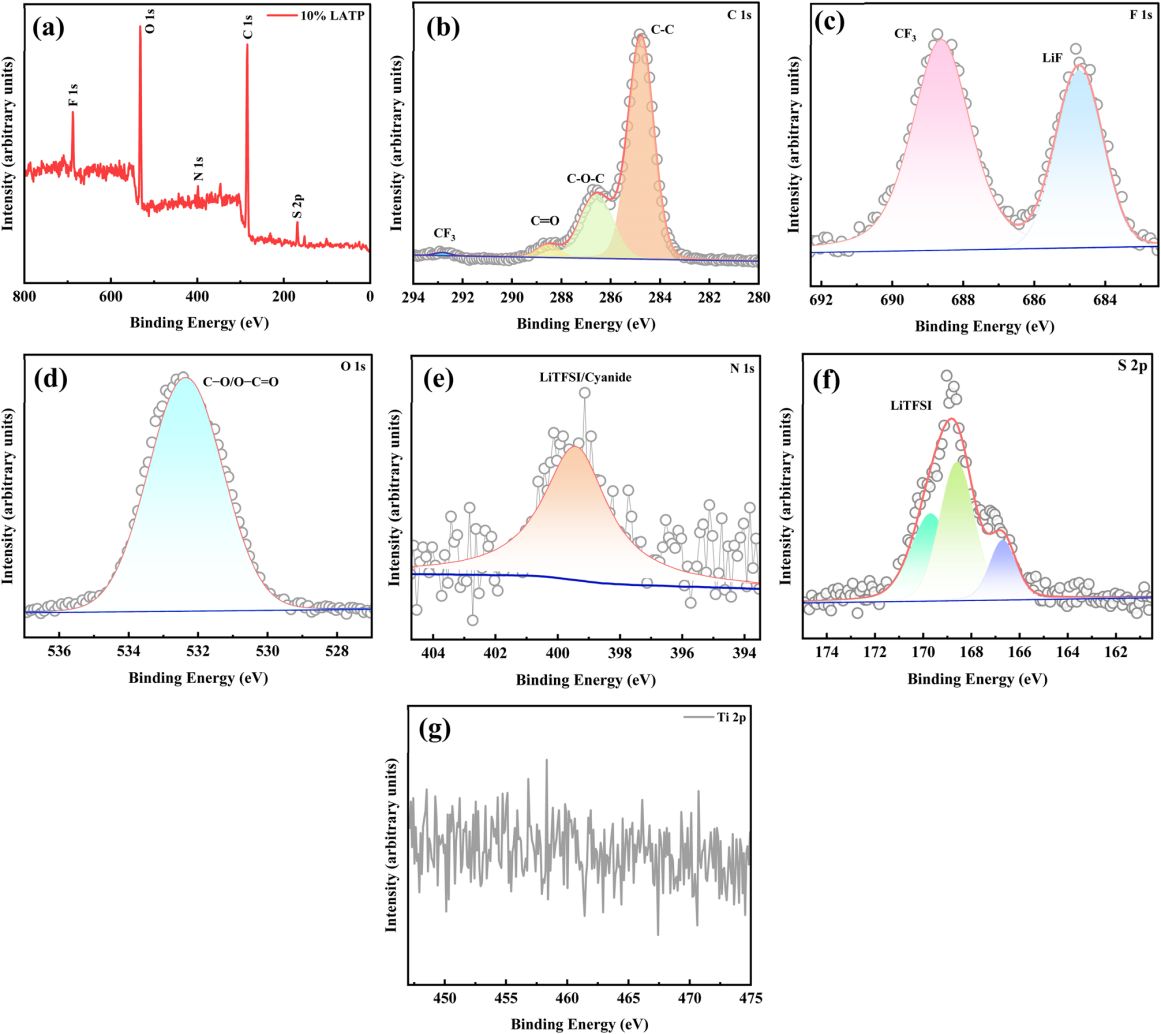
(a) XPS survey spectrum and (b–g) high-resolution spectra of C 1s, F 1s, O 1s, N 1s, S 2p, and Ti 2p, respectively, for the PCSE (10% LATP) membrane.

XPS was employed to examine the chemical interactions within the polymer matrix following the incorporation of the LATP filler. The survey spectrum of the 10% LATP PCSE membrane is shown in Fig. 5. The C 1s spectrum was deconvoluted into peaks corresponding to C–C, C–O–C, C=O, and CF_3_ bonds located at 284.7, 286.5, 288.5, and 292.8 eV, respectively.^44–46^ The F 1s spectrum displays two distinct peaks at 684.7 and 688.6 eV, attributed to LiF and CF_3_ bonding, respectively.^46,47^ The O 1s spectrum shows a characteristic peak at 532.3 eV, associated with C–O/O–C=O bonds.^46,48^ The N 1s spectrum displays a single peak at 399.4 eV, while the S 2p spectrum consists of three peaks at 166.7, 168.5, and 169.7 eV, corresponding to the LiTFSI salt.^46,49^ Notably, no Ti^4+^ signal was detected on the membrane surface, indicating that the surface of the PCSE membrane is composed solely of organic components, with LATP particles embedded within the polymer matrix.^48^

**Fig. 6:**
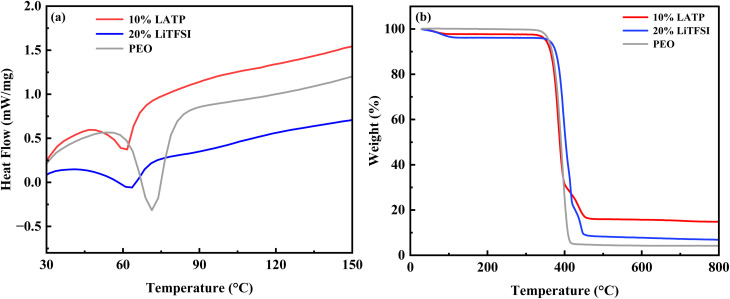
(a) DSC plots, and (b) TGA curves of pure PEO, 20% LiTFSI, and 10% LATP polymer membranes.

The melting temperatures of pure PEO, as well as the 20% LiTFSI and 10% LATP polymer membranes, were obtained from their respective DSC plots shown in Fig. 6(a). For pure PEO, a heat absorption peak is observed around 71 °C, corresponding to its melting temperature. The melting temperatures of the 20% LiTFSI and 10% LATP polymer membranes are 63 °C and 61 °C, respectively. The inclusion of LiTFSI salt and/or LATP ceramic fillers into the PEO polymer matrix lowers the melting temperature of the polymer membranes compared with that of pure PEO.

The thermal stability of the polymer membranes was examined using TGA analysis (Fig. 6(b)). In all cases, a major weight loss occurs between 350–450 °C, corresponding to the decomposition of the PEO polymer.^50^ Further, upon increasing the temperature, no notable weight loss is observed. At 800 °C, the residual weights of the pure PEO, 20% LiTFSI, and 10% LATP polymer membranes are 4.17%, 6.87%, and 14.81% of their initial weights, respectively. This indicates that the 10% LATP PCSE membrane shows better thermal stability compared with both the pure PEO and the 20% LiTFSI polymer membranes.

**Fig. 7:**
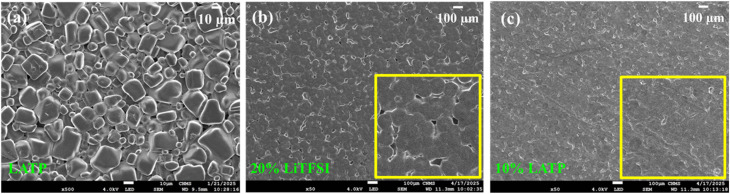
(a) SEM microstructure of the LATP pellet. Top view SEM images of the 20% LiTFSI (b) and 10% LATP. (c) Polymer membranes, with zoomed views shown in the insets.

Fig. 7 (a) displays the SEM microstructural image of the LATP pellet, revealing densely packed and uniform grains. The SEM images of the 20% LiTFSI and 10% LATP polymer membranes are presented in Fig. 7(b) and (c), respectively. The 20% LiTFSI polymer membrane shows several interconnected spheres with visible porosity, likely formed during the evaporation of solvent.^51^ On the other hand, the 10% LATP PCSE membrane depicts smoother surface with more uniformly connected spheres and minimal porosity. Elemental mapping of the SEM cross-sections of 10% LATP PCSE membrane clearly shows the uniform dispersion of LATP fillers throughout the polymer matrix (Fig. S5). The flexible nature of the developed polymer membranes is qualitatively demonstrated by bending and twisting tests, as shown by the digital photographs in Fig. S6.

**Fig. 8:**
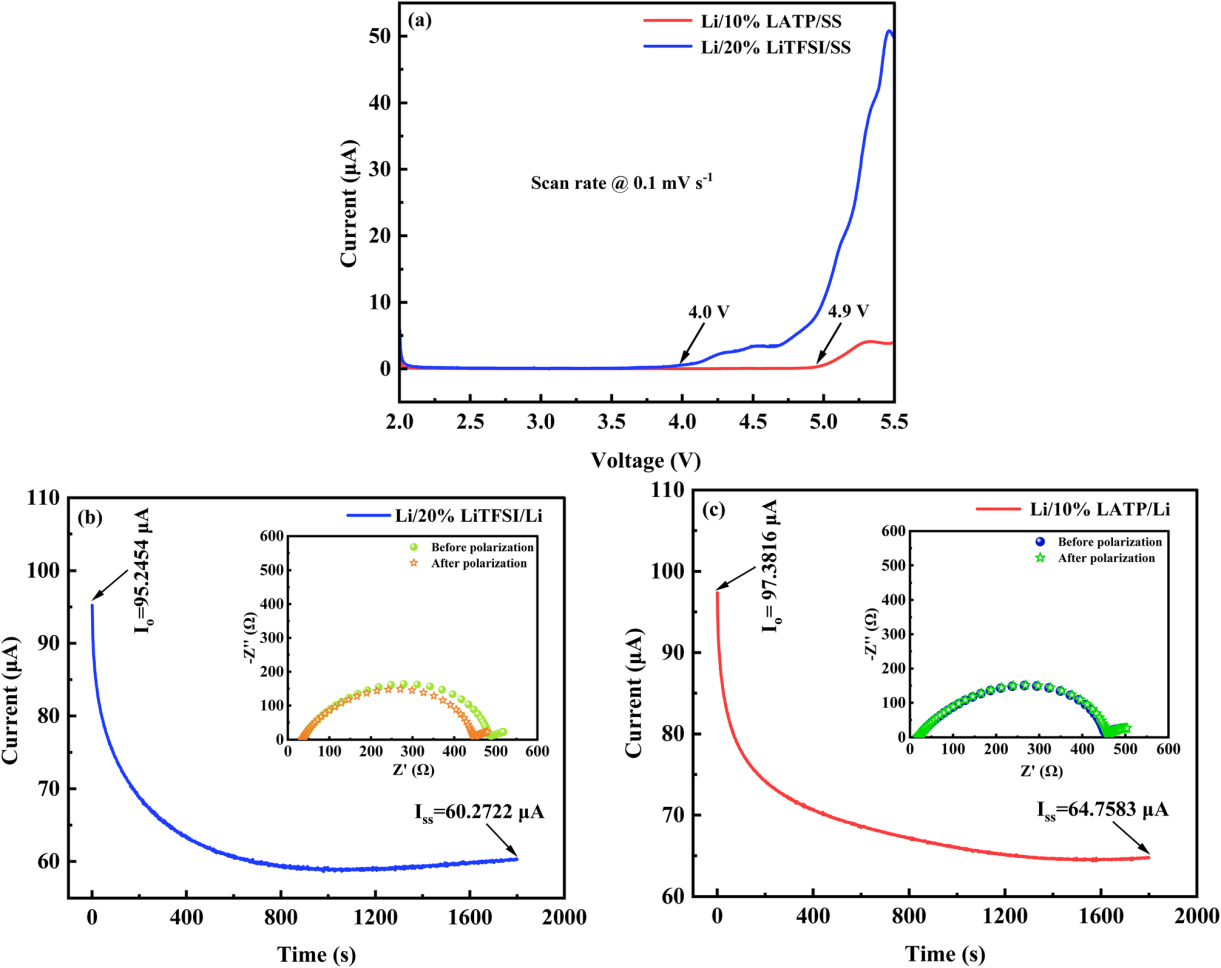
(a) LSV profiles of the 20% LiTFSI and 10% LATP polymer membranes at 60 °C. Current–time response of lithium symmetric (Li/Li) cells employing 20% LiTFSI (b) and 10% LATP (c) polymer membranes under *dc* polarization. Nyquist impedance spectra of the cells acquired before and after *dc* polarization (Insets).

Electrochemical evaluations, including the determination of the electrochemical stability window, Li^+^-ion transference number, and critical current density measurements, are essential prior to further assessing the applicability of the optimized polymer membranes in ASSLBs. The electrochemical stability window determines the voltage limits over which a membrane stays electrochemically stable. Fig. 8(a) shows the LSV plots of the 20% LiTFSI and 10% LATP polymer membranes at 60 °C. Without any sign of decompositions, the electrochemical stability window of the 20% LiTFSI and 10% LATP PCSE polymer membranes were found to be 4.0 V and 4.9 V respectively. The incorporation of 10 wt% LATP into the 20% LiTFSI polymer matrix significantly expands the electrochemical stability window to 4.9 V, making the 10% LATP PCSE membrane more suitable for high-voltage cathode materials. In both cases, an overestimation of approximately 0.3 V arising from the use of an SS/Li configuration cannot be ruled out.

The Li^+^-ion transference number (*t*_Li_^+^) is a measure of an electrolyte's ability to transport Li^+^ -ions.^30^ To determine *t*_Li_^+^, symmetric cells were constructed using lithium-metal electrodes and the polymer membranes as electrolytes. The polarization current *versus* time curves from chronoamperometry, along with the Nyquist plots obtained prior to and after polarization (insets) for the symmetric cells with 20% LiTFSI and 10% LATP polymer membranes are depicted in Fig. 8(b) and (c), respectively. The *t*_Li^+^_ of the polymer membranes were evaluated using the Bruce–Vincent equation (eqn (1)). The *t*_Li^+^_ values for the 20% LiTFSI and 10% LATP polymer membranes were found to be 0.11 and 0.20, respectively, which agree well with previously reported values for similar polymer membrane systems.^23,25^

**Fig. 9:**
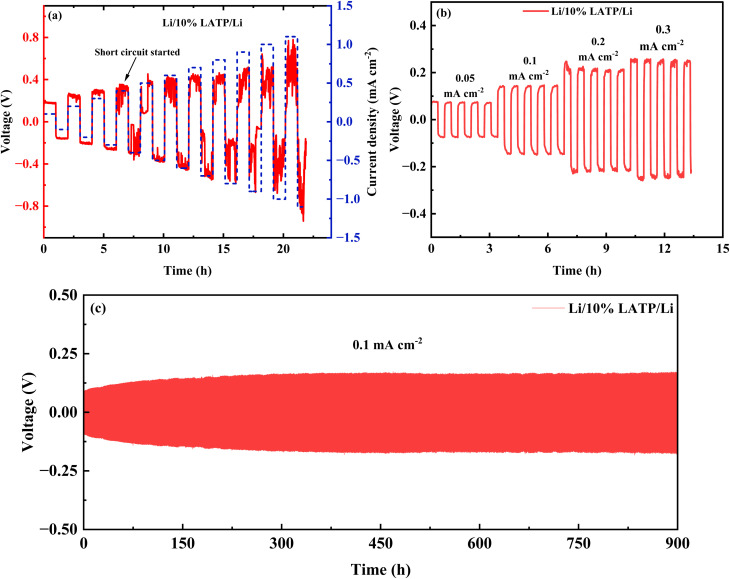
(a) Critical current density analysis, (b) rate performance (0.1 mA cm^−2^–0.3 mA cm^−2^), and (c) cycling stability (0.1 mA cm^−2^) for Li/10% LATP/Li symmetric cells at 60 °C.

The lithium dendrite growth-inhibition performance of the electrolyte was evaluated by measuring its CCD, which corresponds to the minimum current density that induces battery shorting due to dendrite growth. The CCD of the Li/10% LATP/Li symmetric cell was determined by gradually increasing the current density, starting from 0.1 mA cm^−2^ with increments of 0.1 mA cm^−2^ per cycle (Fig. 9(a)). The symmetric cell exhibited stable behavior up to a current density of 0.3 mA cm^−2^, beyond this value short-circuiting was initiated. Accordingly, the CCD of the Li/10% LATP/Li symmetric cell was determined to be 0.3 mA cm^−2^.

The lithium plating/stripping behavior of the Li/10% LATP/Li symmetric cell at various current densities *i.e.* its rate performance (0.05–0.3 mA cm^−2^), is shown in Fig. 9(b). Each voltage plateaus exhibits stable behavior without significant variation. Long-term cycling of the symmetric cell was conducted at 0.1 mA cm^−2^, where it maintained a stable voltage for 900 h, demonstrating notable cycling stability (Fig. 9(c)). The cycling stability of Li/20% LiTFSI/Li symmetric cell is presented in Fig. S7. The Li/20% LiTFSI/Li cell showed sudden voltage instability in the intermediate cycling and gets short-circuited after 180 h of operations. Additionally, The Li/10% LATP/Li symmetric cell showed an initial overpotential of 89.5 mV and a steady-state overpotential of 142.4 mV, whereas the Li/20% LiTFSI/Li symmetric cell exhibited a higher initial overpotential of 190.9 mV, indicating lower electrochemical stability. Thus, the incorporation of LATP powder significantly enhances the interfacial compatibility between the lithium metal and the PCSE membrane interface, resulting in good cycling performance and reduced polarization, which effectively help in the improvement of reversible lithium plating-stripping.

**Fig. 10:**
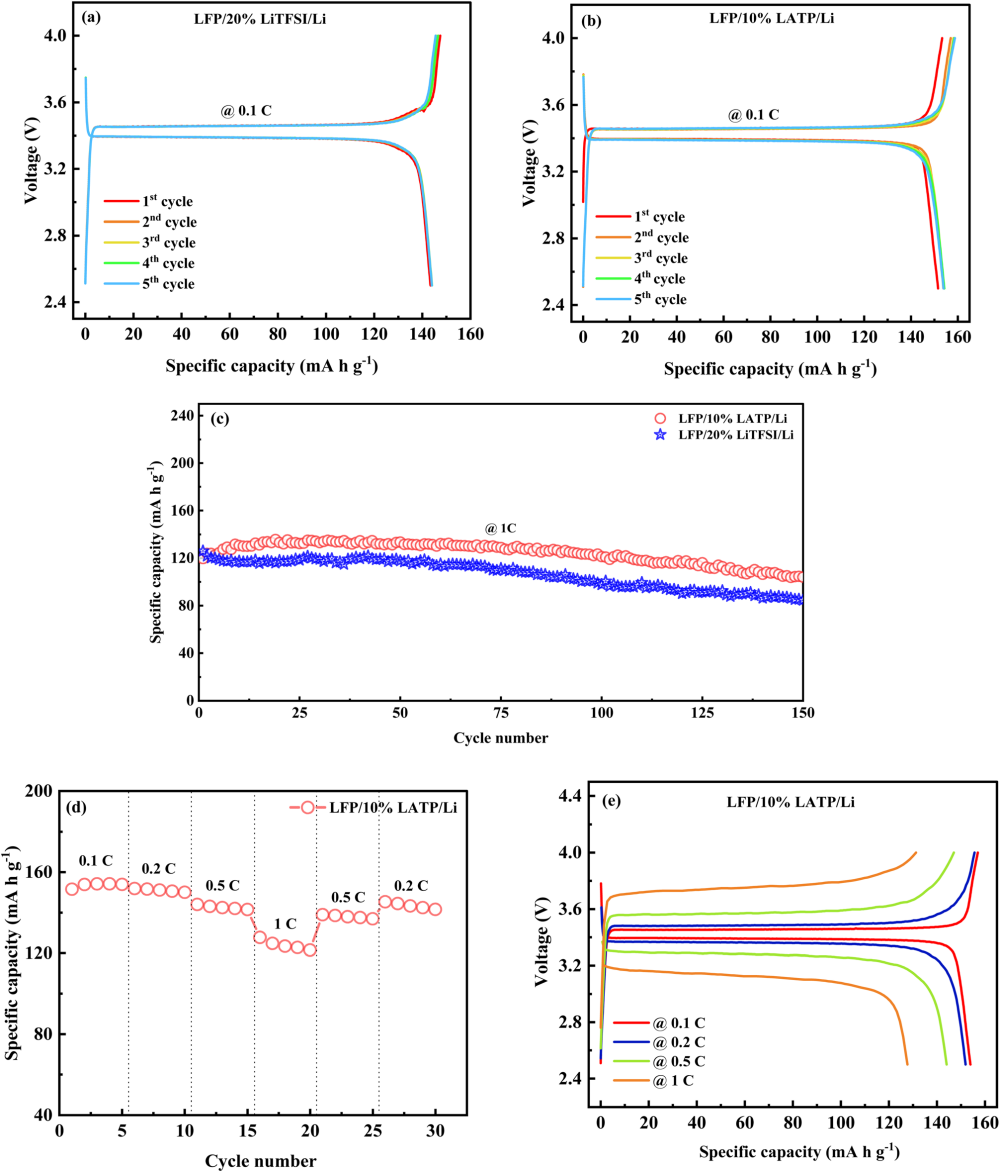
Charge–discharge curves of (a) LFP/20% LiTFSI/Li cell and (b) LFP/10% LATP/Li cell at 0.1C and 60 °C. (c) Cyclic stability of LFP/20% LiTFSI/Li and LFP/10% LATP/Li cells at 1C. (d) Rate performance of the LFP/10% LATP/Li cell. (e) Charge–discharge curves of the LFP/10% LATP/Li cell at various current rates.

To further demonstrate their practical applicability in ASSLBs, polymer membranes containing 20% LiTFSI and 10% LATP were evaluated by fabricating CR2032-type coin cells, with lithium metal anode paired with an LFP cathode. Galvanostatic charge–discharge profiles of LFP/20% LiTFSI/Li and LFP/10% LATP/Li cells at 60 °C are shown in Fig. 10. The LFP/20% LiTFSI/Li cell displays discharge specific capacities of 143.4, 143.9, 144.0, 144.0, and 143.9 mAh g^−1^ from the first to the fifth cycle, respectively, at 0.1C. In comparison, the LFP/10% LATP/Li cell exhibits enhanced discharge specific capacities of 151.6, 153.9, 154.2, 154.2, and 153.9 mAh g^−1^ over the same cycles at 0.1C. Following the 1st cycle, both cells exhibit a slight increase in discharge specific capacity, and from the 3rd cycle onwards, they maintain stable stability, suggesting ongoing chemical and electrochemical activation.^27^

The PCSE membrane with LATP-filler exhibited superior electrochemical performance in comparison to the membrane without the LATP filler at 0.1C. Charge–discharge curves of the LFP/10% LATP/Li cell up to 100 cycles measured at 1C is shown in Fig. S8. The cyclic stability of the cells with 20% LiTFSI and 10% LATP polymer membranes at a constant current rate of 1C is shown in Fig. 10(c). After 150 cycles, the cell with the 20% LiTFSI polymer membrane delivers a discharge-specific capacity of 84.4 mAh g^−1^, with a capacity retention of 66.9%. In comparison, the cell with the 10% LATP PCSE membrane exhibits improved cycling stability, maintaining a higher capacity of 103.9 mAh g^−1^, with an enhanced retention of 86.8%. These results suggest that incorporating LATP as an active filler significantly improves the electrochemical stability of the polymer membrane during prolonged cycling. The enhanced electrochemical characteristics establish 10% LATP PCSE membrane as a favourable electrolyte for ASSLBs. Its rate capability was evaluated under varying current densities. Fig. 10(d) displays the rate performance of the cell with 10% LATP PCSE membrane at different current rates. With an increase in current rate from 0.2C to 1C, the discharge specific capacity decreases from 151.9 to 127.7 mAh g^−1^, resulting in a capacity retention of 84%. Upon returning to 0.2C, the capacity is restored to 145.3 mAh g^−1^, demonstrating superior reversibility and electrochemical stability of the cell. Fig. 10(e) displays the charge–discharge plots of the LFP/10% LATP/Li cell, exhibiting stable discharge-specific capacities of 153.9, 151.9, 144.0, and 127.7 mA g^−1^ at constant currents of 0.1, 0.2, 0.5, and 1C, respectively, indicating good rate capability. The ionic conductivity of the 20% LiTFSI and 10% LATP polymer membranes, as well as the electrochemical performance of the LFP/20% LiTFSI/Li and LFP/10% LATP/Li cells, in comparison with other polymer and PCSE membranes, are summarized in Table S2.

To explore the reason behind the enhanced electrochemical performance of the 10% LATP PCSE membrane compared to the 20% LiTFSI polymer membrane, electrochemical impedance spectroscopy measurements were carried out for the LFP/10% LATP/Li and LFP/20% LiTFSI/Li cells prior to cycling, as shown in Fig. S9. The impedance plots were fitted using the equivalent circuit shown in the inset of the same Fig. S9. In the equivalent circuit, *R*_b_ denotes the electrolyte resistance, while *R*_SEI_ and CPE 1 denote the resistance and constant phase element associated with the SEI layer, contributing to the high-frequency semicircle. *R*_CT_ and CPE 2 denote the resistance and constant phase element related to the charge transfer, forming the semicircle in the medium-frequency region.^52^*W* corresponds to the Warburg impedance, indicating Li^+^-ion diffusion, shown as the sloped line at low frequencies. From the Nyquist plots, it is evident that both *R*_SEI_ and *R*_CT_ are higher for the cell with 20% LiTFSI polymer membrane compared to the cell with the 10% LATP PCSE membrane. The lower interfacial and charge-transfer resistances associated with 10% LATP PCSE membrane account for its improved electrochemical characteristics.

To further explore the potential of the 10% LATP PCSE membrane for the cells with high-voltage cathodes, a NMC622/10% LATP/Li cell was assembled and tested within an electrochemical window of 2.5–4.3 V. The galvanostatic charge–discharge profiles of the NMC622/10% LATP/Li cell at 60 °C are shown in Fig. S10. At a current rate of 0.1C, the NMC622/10% LATP/Li cell delivers discharge-specific capacities of 135.0, 139.6, 140.0, 139.3, and 137.0 mAh g^−1^ from the 1st to 5th cycle, respectively. Consistent with previous observations, the cell exhibits a modest increase in discharge capacity after the initial cycle, which stabilizes from the third cycle onwards. Fig. S10 also presents the charge–discharge curves of the NMC622/10% LATP/Li cell, exhibiting discharge specific capacities of 139.6 and 119.9 mAh g^−1^ at constant current rates of 0.1 and 0.2C, respectively. These findings highlight the superior electrochemical performance of the 10% LATP PCSE membrane for applications in high-voltage ASSLBs.

## Conclusions

This study investigated the influence of NASICON-type LATP fillers, crystallizing in a rhombohedral structure, on the structural and transport properties of PEO-LiTFSI polymer membranes. Among the PCSE membranes studied, the PEO-LiTFSI polymer membrane with 10 wt% LATP fillers (10% LATP) exhibited a wider electrochemical stability window and an improved Li^+^-ion transference number. The 10% LATP PCSE membrane demonstrated a favourable ionic conductivity of 0.19 × 10^−3^ S cm^−1^ and a wider electrochemical stability window of 4.9 V at 60 °C. In addition, it showed a lower melting temperature and better thermal stability compared to the filler-free polymer membranes. The 10% LATP PCSE membrane was found to remain electrochemically stable for over 900 h, compared to 180 h for the 20% LiTFSI polymer membrane. The applicability of these polymer membranes in ASSLBs was demonstrated using both the commercially viable LFP and the high-voltage NMC622 cathodes. The LFP/10%LATP/Li cell suggested superior rate capability and durable cycling, delivering 103.9 mAh g^−1^ at 1C with 86.8% capacity retention after 150 cycles.

## Author contributions

Sumit Khatua: investigation, methodology, formal analysis, writing – original draft. Sasikumar K: methodology. K. Ramakrushna Achary: methodology. Gajjala Sindhu: methodology. Tausif Alam: writing – review & editing. L. N. Patro: conceptualization, writing – review & editing, project administration, supervision.

## Conflicts of interest

The authors declare no competing financial interest.

## Supplementary Material

RA-016-D5RA09944G-s001

## Data Availability

Any data that support the finding of this study are included within the article, and all the data used in the manuscript are available. Supplementary information (SI) is available. See DOI: https://doi.org/10.1039/d5ra09944g.

## References

[cit1] Kim T., Song W., Son D., Ono L. K., Qi Y. (2019). Lithium-ion batteries: Outlook on present, future, and hybridized technologies. J. Mater. Chem. A.

[cit2] Goodenough J. B., Kim Y. (2010). Challenges for rechargeable Li batteries. Chem. Mater..

[cit3] Khatua S., Achary K. R., Rao Y. B., Sasikumar K., Samal A. K., Patro L. N. (2024). Physicochemical activation of soap-nut seeds-derived hard carbon as a sustainable anode for lithium-ion batteries. New J. Chem..

[cit4] Goodenough J. B., Park K. (2013). The Li-ion rechargeable battery: A perspective. J. Am. Chem. Soc..

[cit5] Zhao Q., Stalin S., Zhao C., Archer L. A. (2020). Designing solid-state electrolytes for safe, energy-dense batteries. Nat. Rev. Mater..

[cit6] Khatua S., Rao Y. B., Achary K. R., Patro L. N. (2023). Li-ion transport studies of NASICON- type LiZr_2_(PO_4_)_3_ solid electrolyte crystallizing in rhombohedral structure at room temperature. Surf. Interfaces.

[cit7] Takada K. (2018). Progress in solid electrolytes toward realizing solid-state lithium batteries. J. Power Sources.

[cit8] Nzereogu P. U., Oyesanya A., Ogba S. N., Ayanwunmi S. O., Sobajo M. S., Chimsunum V. C., Ayanwunmi V. O., Amoo M. O., Adefemi O. T., Chukwudi C. C. (2025). Solid-state lithium-ion battery electrolytes: Revolutionizing energy density and safety. Hybrid Adv..

[cit9] Hou M., Liang F., Chen K., Dai Y., Xue D. (2020). Challenges and perspectives of NASICON-type solid electrolytes for all solid-state lithium batteries. Nanotechnology.

[cit10] Chan C. H., Wong H. H., Liang S., Sun M., Wu T., Lu Q., Lu L., Chen B., Huang B. (2024). Electrolyte developments for all-solid-state lithium batteries: Classifications, recent advances and synthesis methods. Batter. Supercaps.

[cit11] Ding Z., Li J., Li J., An C. (2020). Review- Interfaces: Key issue to be solved for all solid-state lithium battery technologies. J. Electrochem. Soc..

[cit12] Lee T., Joo S., Kang S., Kim T., Park Y., Chae Y., Kim K., Cho W., Kim S. (2025). Multi-solid-electrolyte systems for all-solid-state batteries: Current status and future prospects. ACS Appl. Energy Mater..

[cit13] Knauth P. (2009). Inorganic solid Li ion conductors: An overview. Solid State Ionics.

[cit14] Umair M., Zhou S., Li W., Rana H. T. H., Yang J., Cheng L., Li M., Yu S., Wei J. (2025). Oxide solid electrolytes in solid-state batteries. Batter. Supercaps.

[cit15] Zhang Z., Shao Y., Lotsch B., Hu Y., Li H., Janek J., Nazar L. F., Nan C., Maier J., Armand M., Chen L. (2018). New horizons for inorganic solid state ion conductors. Energy Environ. Sci..

[cit16] Khatua S. , Rao Y. B., Achary K. R., Manne V. and Patro L. N., NASICON-Type Li-Ion Conducting Solid Electrolytes for All-Solid-State Li-Ion Batteries, Electrolytes for Energy Storage Applications. CRC Press, Boca Raton, 2024, pp. 112–131, 10.1201/9781003441182

[cit17] He L., Liang W., Cao J., Wu D. (2022). PI-LATP-PEO electrolyte with high safety performance in solid-state lithium metal batteries. ACS Appl. Energy Mater..

[cit18] Khatua S., Achary K. R., Sasikumar K., Korlapati L. H., Patro L. N. (2026). Engineering dense superionic Li_1+x_Al_x_Ti_2-x_(PO_4_)_3_ solid electrolytes for safer solid-state Li-ion batteries: Impact of sintering temperature and Al^3+^ doping. Solid State Ionics.

[cit19] Yang Z., Yuan H., Zhou C., Wu Y., Tang W., Sang S., Liu H. (2020). Facile interfacial adhesion enabled LATP-based solid-state lithium metal battery. Chem. Eng. J..

[cit20] Yu X., Manthiram A. (2020). A long cycle life, all-solid-state lithium battery with a ceramic-polymer composite electrolyte. ACS Appl. Energy Mater..

[cit21] Yu X., Li J., Manthiram A. (2020). Rational design of a laminated dual-polymer/polymer-ceramic composite electrolyte for high-voltage all-solid-state lithium batteries. ACS Mater. Lett..

[cit22] Li S., Zhang S., Shen L., Liu Q., Ma J., Lv W., He Y., Yang Q. (2020). Progress and perspective of ceramic/polymer composite solid electrolytes for lithium batteries. Adv. Sci..

[cit23] Ban X., Zhang W., Chen N., Sun C. (2018). A high-performance and durable poly(ethylene oxide)-based composite solid electrolyte for all solid-state lithium battery. J. Phys. Chem. C.

[cit24] Zhao E., Guo Y., Xin Y., Xu G., Guo X. (2020). Enhanced electrochemical properties and interfacial stability of poly(ethylene oxide) solid electrolyte incorporating nanostructured Li_1.3_Al_0.3_Ti_1.7_(PO_4_)_3_ fillers for all solid state lithium ion batteries. Int. J. Energy Res..

[cit25] Dong G., Mao Y., Yang G., Li Y., Song S., Xu C., Huang P., Hu N., Fu S. (2021). High-strength poly(ethylene oxide) composite electrolyte reinforced with glass fiber and ceramic electrolyte simultaneously for structural energy storage. ACS Appl. Energy Mater..

[cit26] Nkosi F. P., Cuevas I., Valvo M., Mindemark J., Mahun A., Abbrent S., Brus J., Kobera L., Edström K. (2024). Understanding lithium-ion conductivity in NASICON-type polymer- in-ceramic composite electrolytes. ACS Appl. Energy Mater..

[cit27] Li S., Lu J., Geng Z., Chen Y., Yu X., He M., Li H. (2022). Solid polymer electrolyte reinforced with a Li_1.3_Al_0.3_Ti_1.7_(PO_4_)_3_-coated separator for all-solid-state lithium batteries. ACS Appl. Mater. Interfaces.

[cit28] Chen H., Zhou C., Dong X., Yan M., Liang J., Xin S., Wu X., Guo Y., Zeng X. (2021). Revealing the superiority of fast ion conductor in composite electrolyte for dendrite-free lithium-metal batteries. ACS Appl. Mater. Interfaces.

[cit29] Zou Y., Weng H., Jiang Z., Wang C., Zhao N., Li J., Chen X., Mei Y. (2024). Aqueous tape casting phosphate ceramic electrolyte membrane for high performance
all solid-state lithium metal battery. J. Power Sources.

[cit30] Liu L., Zhang D., Zhao J., Shen J., Li F., Yang Y., Liu Z., He W., Zhao W., Liu J. (2022). Synergistic effect of lithium salts with fillers and solvents in composite electrolytes for superior room-temperature solid-state lithium batteries. ACS Appl. Energy Mater..

[cit31] Guo Y., Zhao E., Su W., Liu Z., Li J. (2025). One-dimensional LATP nanofiber reinforced PEO solid composite electrolyte for all-solid-state lithium-ion batteries with excellent cycling performance. Chem. Eng. J..

[cit32] Méry A., Rousselot S., Lepage D., Dollé M. (2021). A critical review for an accurate electrochemical stability window measurement of solid polymer and composite electrolytes. Materials.

[cit33] Dashjav E., Ma Q., Xu Q., Tsai C., Giarola M., Mariotto G., Tietz F. (2018). The influence of water on the electrical conductivity of aluminum-substituted lithium titanium phosphates. Solid State Ionics.

[cit34] Chandran C. V., Pristat S., Witt E., Tietz F., Heitjans P. (2016). Solid-state NMR investigations on the structure and dynamics of the ionic conductor Li_1+x_Al_x_Ti_2-x_(PO_4_)_3_ (0.0 ≤ x ≤ 1.0). J. Phys. Chem. C.

[cit35] Achary K. R., Khatua S., Bharathi K. K., Patro L. N. (2024). TlSn_2_F_5_, a SnF_2_-based solid electrolyte with high ionic conductivity and electrochemical stability for all-solid-state fluoride ion batteries. Dalton Trans..

[cit36] Butzelaar A. J., Liu K. L., Röring P., Brunklaus G., Winter M., Theato P. (2021). A systematic study of vinyl ether-based poly(ethylene oxide) side-chain polymer electrolytes. ACS Appl. Polym. Mater..

[cit37] Fang C., Huang Y., Sun Y., Sun P., Li K., Yao S., Zhang M., Fang W., Chen J. (2024). Revealing and reconstructing the 3D Li-ion transportation network for superionic poly(ethylene) oxide conductor. Nat. Commun..

[cit38] Kim K., Kuhn L., Alabugin I. V., Hallinan Jr D. T. (2020). Lithium salt dissociation in diblock copolymer electrolyte using fourier transform infrared spectroscopy. Front. Energy Res..

[cit39] Vélez J. F., Aparicio M., Mosa J. (2016). Covalent silica-PEO-LiTFSI hybrid solid electrolytes via sol-gel for Li-ion battery applications. Electrochim. Acta.

[cit40] Zhang L., Feng J., Zhu G., Yan J., Bartlett S., Wang Z., Hao Z., Gao Z., Wang R. (2024). Effect of Li_6.4_La_3_Zr_1.4_Ta_0.6_O_12_ fillers on the interfacial properties between composite PEO-LiTFSI electrolytes with Li metal during cycling. ACS Appl. Mater. Interfaces.

[cit41] Banitaba S. N., Semnani D., Fakhrali A., Ebadi S. V., Heydari-soureshjani E., Rezaei B., Ensafi A. A. (2020). Electrospun PEO nanofibrous membrane enable by LiCl, LiClO_4_, and LiTFSI salts: A versatile solvent-free electrolyte for lithium-ion battery application. Ionics.

[cit42] Alam T., Mondal A., Das A. (2024). Role of LLZO dispersion in ion migration property of a ceramic integrated polymer composite electrolyte. Ionics.

[cit43] Rey I., Lassègues J. C., Grondin J., Servant L. (1998). Infrared and Raman study of the PEO-LiTFSI polymer electrolyte. Electrochim. Acta.

[cit44] Wurster V., Engel C., Graebe H., Ferber T., Jaegermann W., Hausbrand R. (2019). Characterization of the interfaces in LiFePO_4_/PEO-LiTFSI composite cathodes and to the adjacent layers. J. Electrochem. Soc..

[cit45] Simon F. J., Hanauer M., Richter F. H., Janek J. (2020). Interphase formation of PEO_20_:LiTFSI-Li_6_PS_5_Cl composite electrolytes with lithium metal. ACS Appl. Mater. Interfaces.

[cit46] Breuer O., Gofer Y., Elias Y., Fayena-Greenstein M., Aurbach D. (2024). Misuse of XPS in analyzing solid polymer electrolytes for lithium batteries. J. Electrochem. Soc..

[cit47] Yusim Y., Moryson Y., Seipp K., Sann J., Henss A. (2024). Challenges in XPS analysis of PEO-LiTFSI-based solid electrolytes: How to overcome X-ray-induced photodecomposition. Batter. Supercaps.

[cit48] Li S., Sun G., He M., Li H. (2022). Organic-inorganic composite electrolytes optimized with fluoroethylene carbonate additive for quasi-solid-state lithium-metal batteries. ACS Appl. Mater. Interfaces.

[cit49] Xu C., Sun B., Gustafsson T., Edström K., Brandell D., Hahlin M. (2014). Interface layer formation in solid polymer electrolyte lithium batteries: An XPS study. J. Mater. Chem. A.

[cit50] Yao Z., Zhu K., Li X., Zhang J., Li J., Wang J., Yan K., Liu J. (2021). Double-layered multifunctional composite electrolytes for high-voltage solid-state lithium-metal batteries. ACS Appl. Mater. Interfaces.

[cit51] Ock J., Fujishiro M., Ueno K., Kawamura I., Tatara R., Hashimoto K., Watanabe M., Dokko K. (2021). Transport properties of flexible composite electrolytes composed of Li_1.5_Al_0.5_Ti_1.5_(PO_4_)_3_ and a poly(vinylidene fluoride-co hexafluoropropylene) gel containing a highly concentrated Li[N(SO_2_CF_3_)_2_]/sulfolane electrolyte. ACS Omega.

[cit52] Pham T. D., Lee K. (2021). Simultaneous stabilization of the solid/cathode electrolyte interface in lithium metal batteries by a new weakly solvating electrolyte. Small.

