# Supplementary material for: Flexible and stable PEO-based polymer composite solid electrolyte membranes incorporating NASICON-type Li1.3Al0.3Ti1.7(PO4)3 for high-performance all-solid-state lithium batteries
Source: RSC Adv. 2026 Mar 5;16(14):12590–603. doi: 10.1039/d5ra09944g (PMC12962239; doi:10.1039/d5ra09944g)
Supplement: RA-016-D5RA09944G-s001 [file RA-016-D5RA09944G-s001.pdf]

Supplementary Information (SI) for RSC Advances.
This journal is © The Royal Society of Chemistry 2026

Supporting Information for

Flexible and Stable PEO-Based Polymer Composite Solid Electrolyte Membranes Incorporating NASICON-Type $Li_{1.3}Al_{0.3}Ti_{1.7}(PO_4)_3$ for High-Performance All-Solid-State Lithium Batteries

Sumit Khatua,[1,2] Sasikumar K,[1,2] K. Ramakrushna Achary,[3] Gajjala Sindhu,[1] Tausif Alam,[1] L. N. Patro[1,2*]

[1]Solid State Ionics Lab, Department of Physics, SRM University AP, Amaravati, 522240, Andhra Pradesh, India

[2]SRM-Amara Raja Center for Energy Storage Devices, SRM University AP, Amaravati, 522240, Andhra Pradesh, India

[3]Department of Chemistry, SRM University AP, Amaravati, 522240, Andhra Pradesh, India

---

[*]**Corresponding author:** laxminarayana.p@srmap.edu.in; patrolng@gmail.com (L. N. Patro)

Tel: 0863-2343000

Fax: 0863-2343111

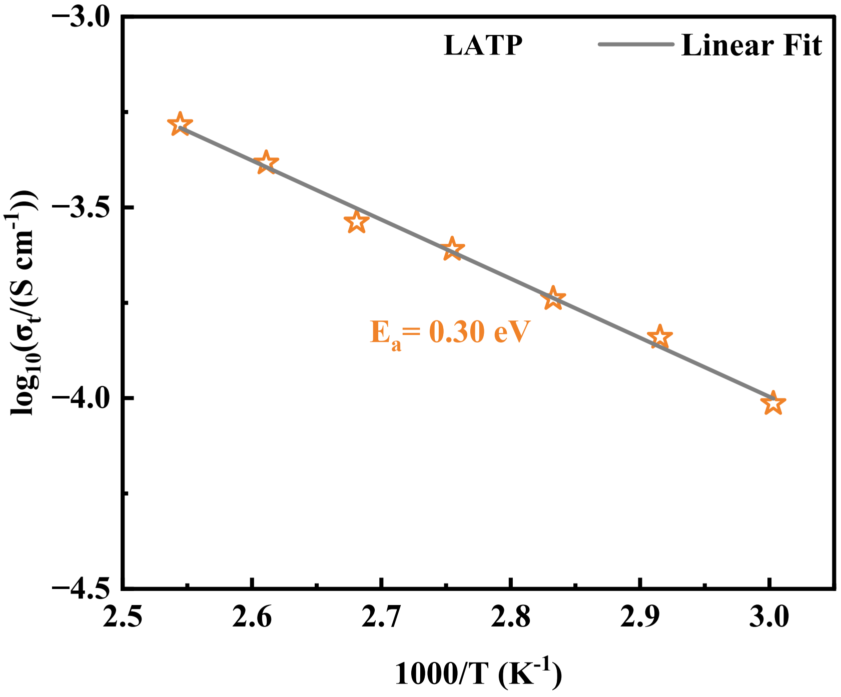

Fig. S1. Temperature dependent conductivity plot of LATP pellet.

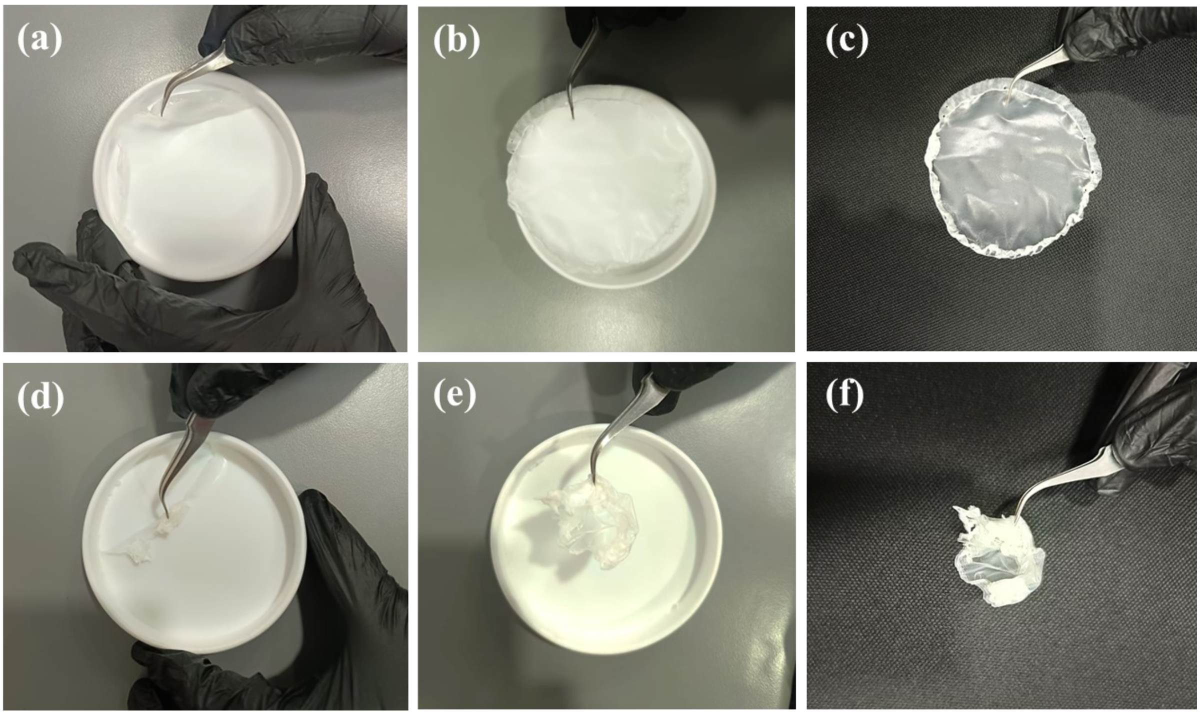

Fig. S2 (a-c). Photographs of the 20% LiTFSI and **(d-f).** 25% LiTFSI polymer membranes at RT during their development.

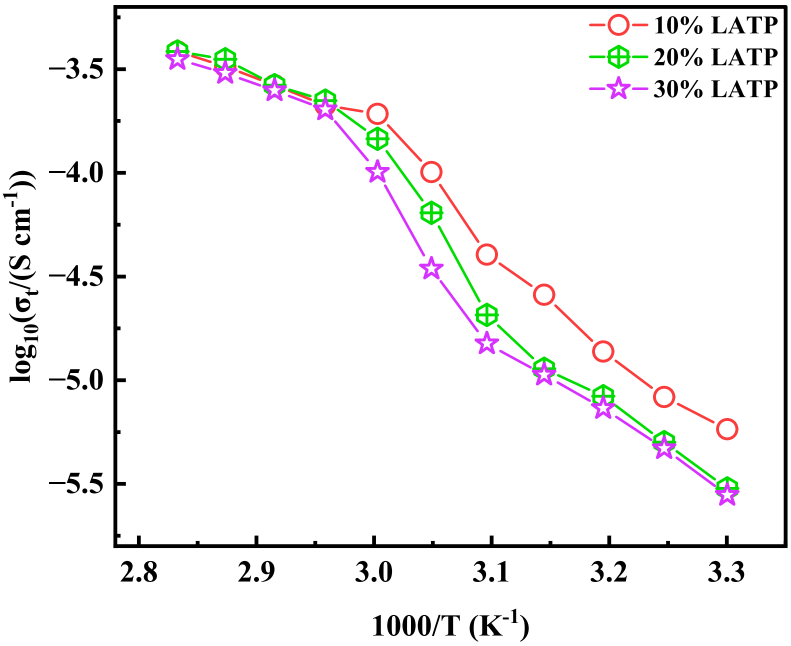

Fig S3. Temperature-dependent ionic conductivity (30-80°C) of PCSE membranes with varying LATP ceramic filler concentrations.

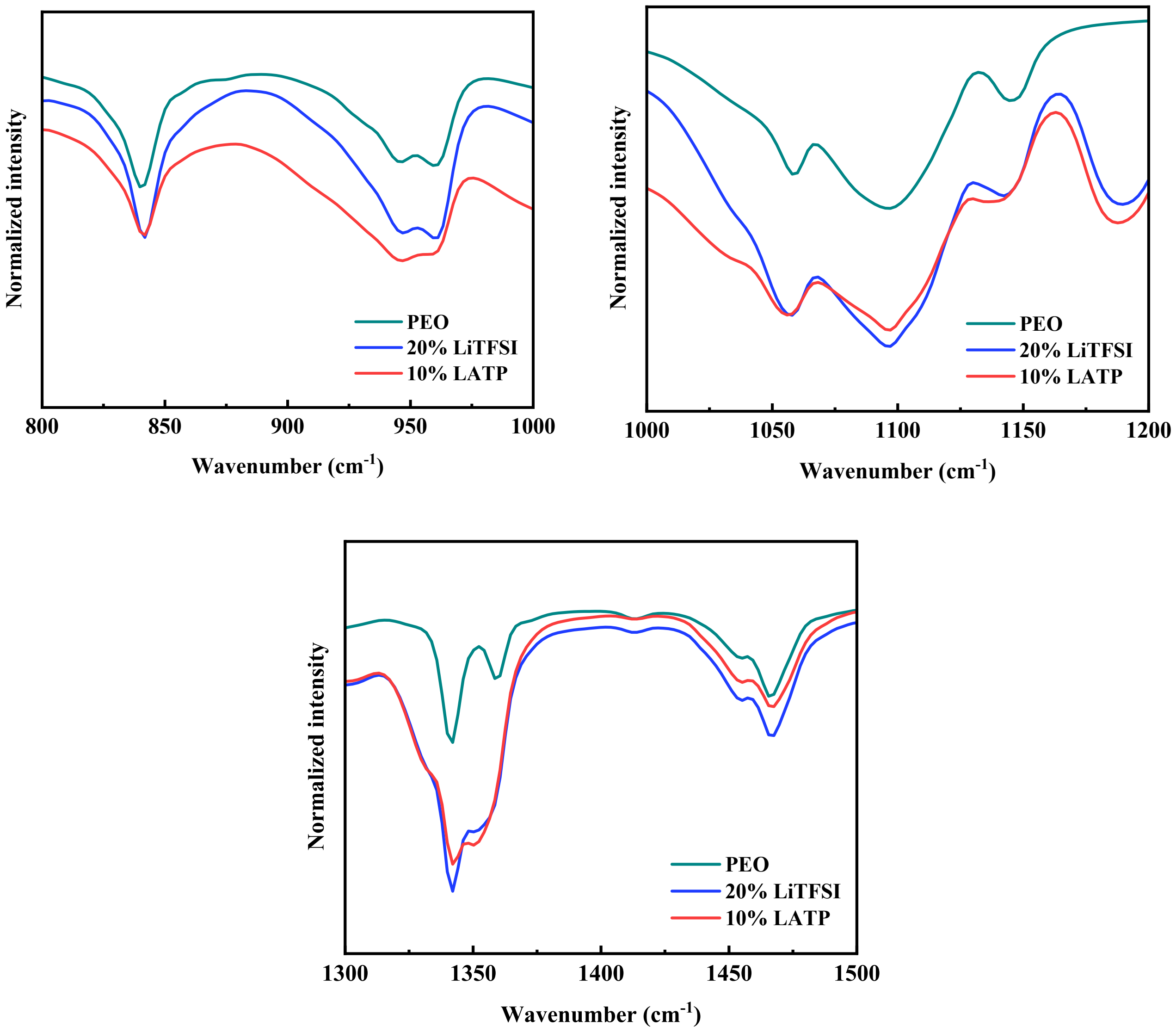

Fig. S4. Magnified regions of the FTIR spectra at specific wavenumber ranges.

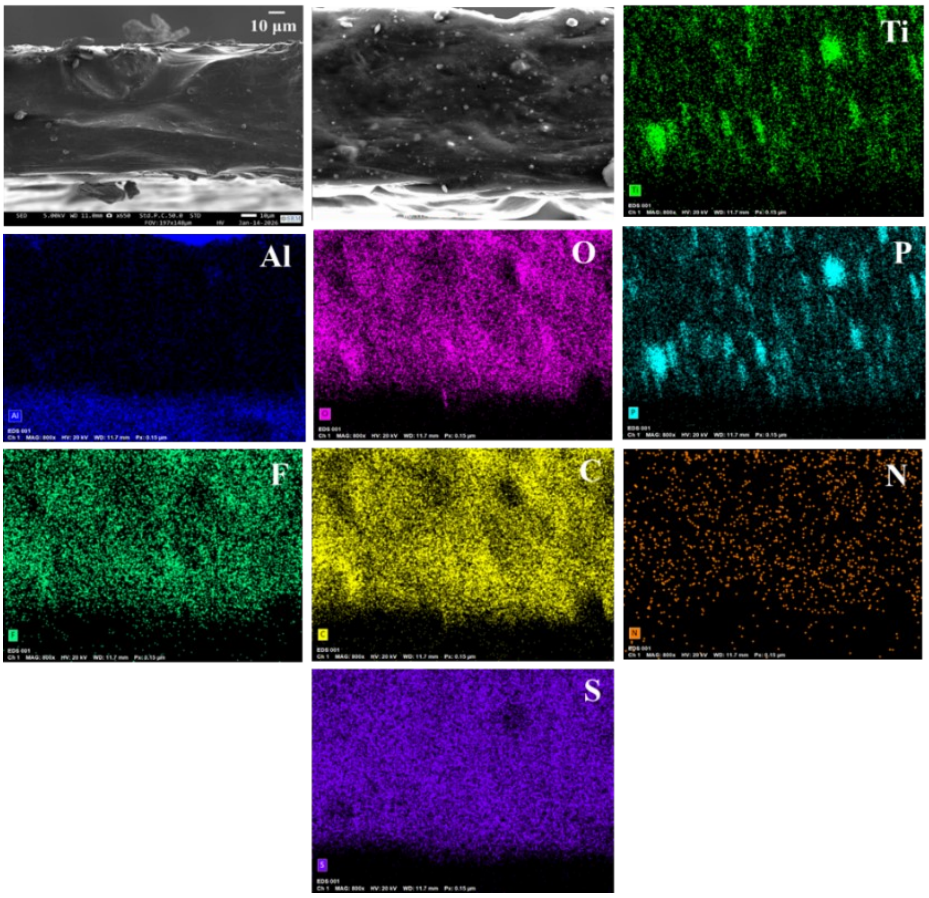

Fig S5. Cross-sectional SEM image with corresponding elemental mapping of the 10% LATP PCSE membrane.

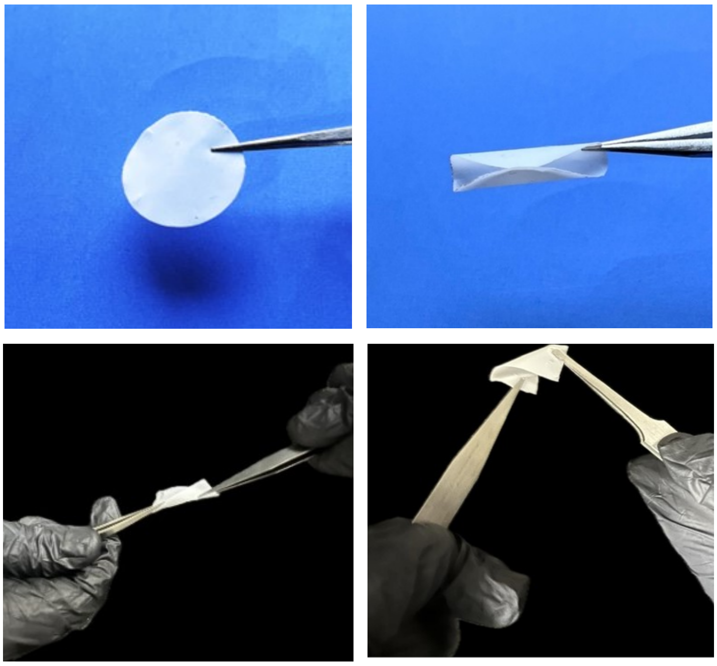

Fig S6. Digital photographs showing the flexibility of the 10% LATP PCSE membrane under bending and twisting tests.

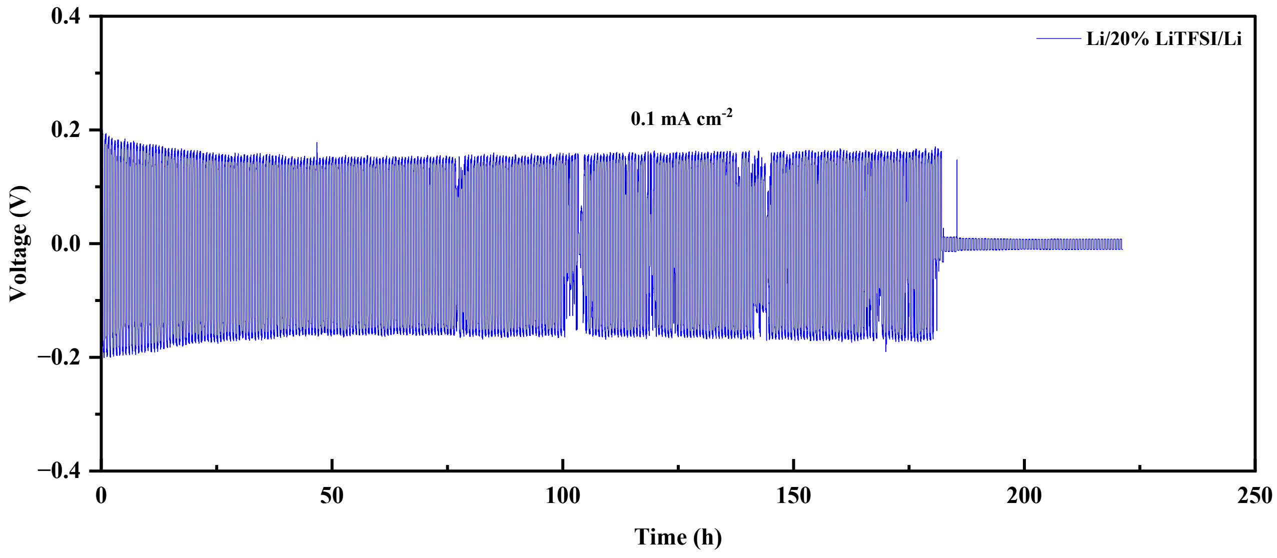

Fig. S7. Cycling stability of the Li/20% LiTFSI/ Li symmetric cell during lithium stripping-plating at a constant current density of 0.1 mA cm^{-2}.

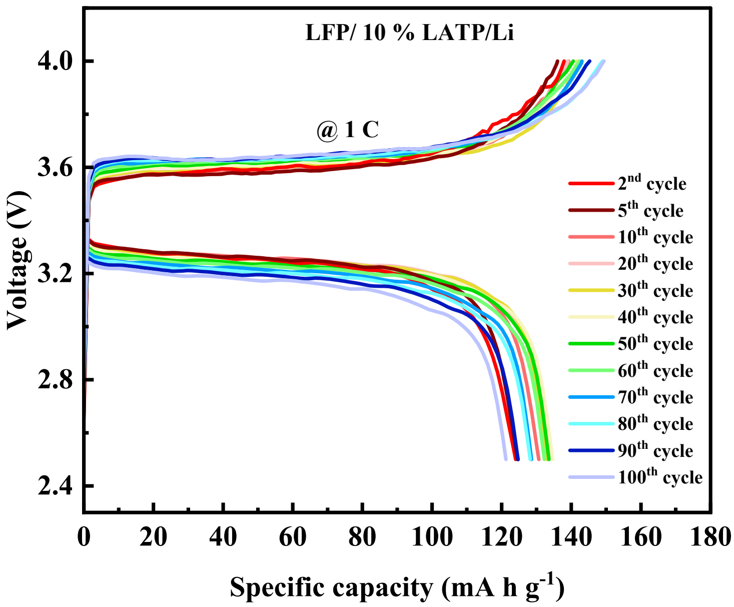

Fig. S8. Galvanostatic charge-discharge profiles showing the cycling performance of LFP/10% LATP/Li cell at 1 C.

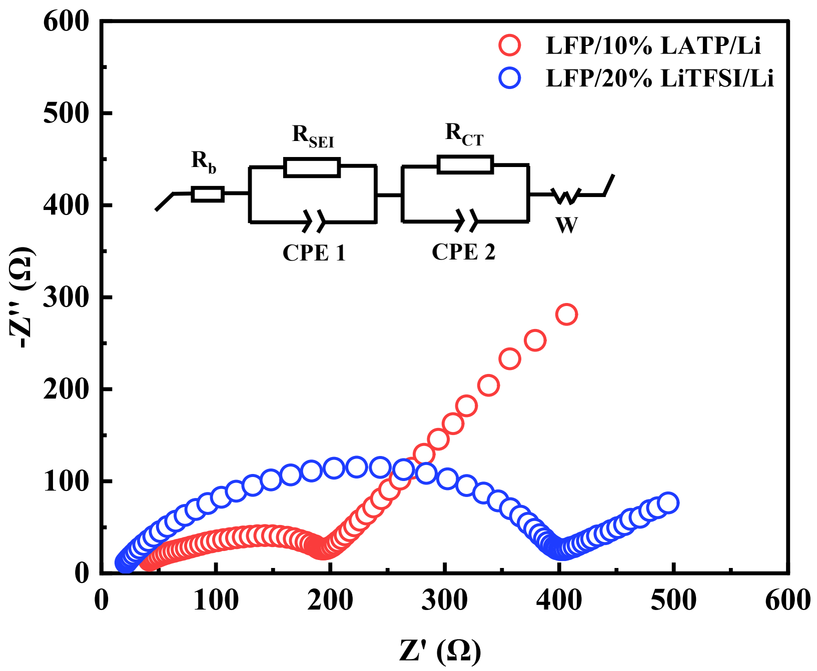

Fig. S9. Nyquist plots of the LFP/PCSE/Li cell before cycling at 60°C.

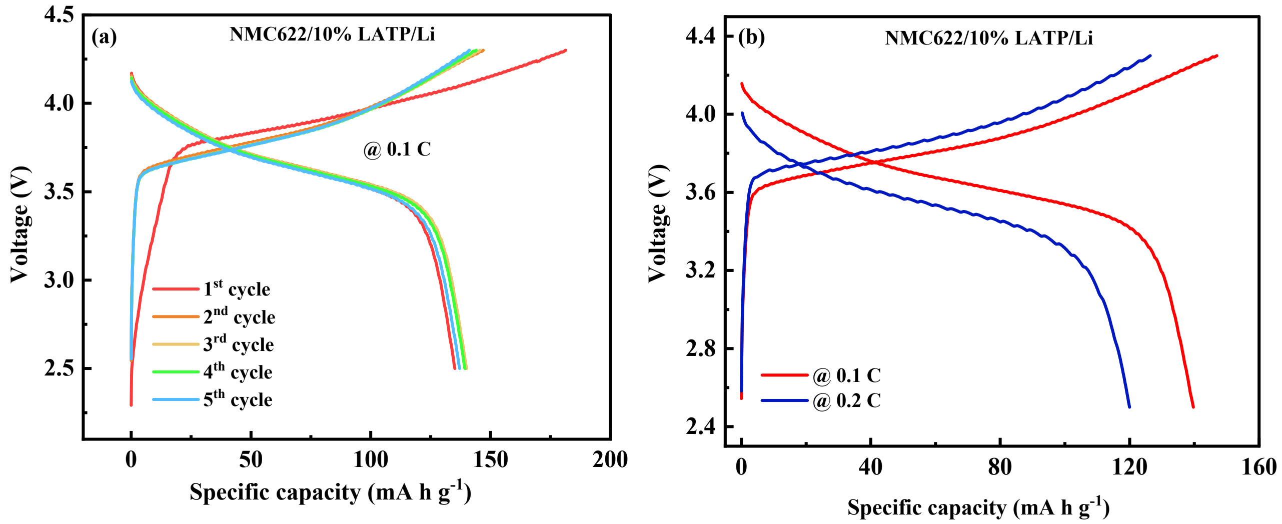

Fig. S10(a) Charge-discharge profiles of NMC622/10% LATP/Li cell at 0.1 C. **(b)** Charge-discharge curves of NMC622/10% LATP/Li cell at two different current rates.

Table S1: Conductivity values of different PEO-based polymer membranes.

Sample name	Conductivity (RT) ($S\ cm^{-1}$)	Conductivity (60°C) ($S\ cm^{-1}$)
15% LiTFSI	0.13×10^{-5}	0.30×10^{-4}
20% LiTFSI	0.28×10^{-5}	0.22×10^{-3}
10% LATP	0.34×10^{-5}	0.19×10^{-3}
20% LATP	0.26×10^{-5}	0.81×10^{-4}
30% LATP	0.22×10^{-5}	0.49×10^{-4}

Table S2. Comparison of the ionic conductivity and electrochemical performance of 20% LiTFSI and 10% LATP polymer membranes with previously reported values for polymer and composite polymer electrolytes.

**Polymer Electrolyte**	**Ionic conductivity ($S\ cm^{-1}$)**	**Discharge capacity ($mA\ h\ g^{-1}$) $LiFePO_4$//Li**	**Current rate**	**Reference**
PEO-LITFSI	2.95×10^{-4} at 55°C	151.6 at 55°C	0.2 C	**1**
PEO-LITFSI	2.86×10^{-4} at 60°C	152.02 at 60°C	0.2 C	**2**
PVDF−HFP- LiTFSI	7.1×10^{-5} at RT	112 at RT	0.2 C	**3**
PVDF–HFP-LiTFSI	2.5×10^{-4} at 25°C	127.4 at 25°C	0.1 C	**4**
PEO-LiTFSI (20 % LiTFSI)	2.2×10^{-4} at 60°C	143.4 at 60°C	0.1 C	This work
PEO-$LiClO_4$-LATP	1.6×10^{-3} at 80°C	130.2 at 80°C	0.2 C	**5**
PEO-LiTFSI-LATP-FEC	1.99×10^{-4} at 30°C	147 at RT	0.1 C	**6**
PEO-LiTFSI-LAGP	1.6×10^{-5} at 20°C	166 at 80°C	0.1 C	**7**
PEO-LiTFSI-LLZTO	2.12×10^{-4} at 60°C	153.8 at 60°C	0.3 C	**8**

PVDF-LiTFSI-LATP	2.44×10^{-4} at 25°C	155 at 30°C	0.2 C	**9**
PEO-LiTFSI-LATP (10% LATP)	1.9×10^{-4} at 60°C	151.6 at 60°C	0.1 C	This work

References

1. E. Zhao, Y. Guo, Y. Xin, G. Xu, X. Guo, Enhanced electrochemical properties and interfacial stability of poly(ethylene oxide) solid electrolyte incorporating nanostructured $Li_{1.3}Al_{0.3}Ti_{1.7}(PO_4)_3$ fillers for all solid state lithium ion batteries. Int. J. Energy Res., 2020, **45**, 6876-6887.
   https://doi.org/10.1002/er.6278
2. Y. Guo, E. Zhao, W. Su, Z. Liu, J. Li, One-dimensional LATP nanofiber reinforced PEO solid composite electrolyte for all-solid-state lithium-ion batteries with excellent cycling performance. Chem. Eng. J., 2025, **511**, 162127.
   https://doi.org/10.1016/j.cej.2025.162127
3. Y. Li, H. Wang, Composite solid electrolytes with NASICON-type LATP and PVdF–HFP for solid-state lithium batteries. Ind. Eng. Chem. Res., 2021, **60**, 1494-1500.
   https://dx.doi.org/10.1021/acs.iecr.0c05075
4. C. Zhao, W. Wei, Z. Li, Z. Liu, Optimised sol-gel synthesis of Al-doped LLZO/PVDF–HFP/LiTFSI composite electrolytes with enhanced electrochemical performance. Ceram. Int., 2025, **51**, 57807-57818.
   https://doi.org/10.1016/j.ceramint.2025.09.481
5. X. Ban, W. Zhang, N. Chen, C. Sun, A high-performance and durable poly(ethylene oxide) based composite solid electrolyte for all solid-state lithium battery. J. Phys. Chem. C, 2018, **122**, 9852-9858.

https://doi.org/10.1021/acs.jpcc.8b02556

6. S. Li, G. Sun, M. He, H. Li, Organic-inorganic composite electrolytes optimized with fluoroethylene carbonate additive for quasi-solid-state lithium-metal batteries. ACS Appl. Mater. Interfaces, 2022, **14**, 20962-20971.
https://doi.org/10.1021/acsami.2c02038
7. G. Piana, F. Bella, F. Geobaldo, G. Meligrana, C. Gerbaldi, PEO/LAGP hybrid solid polymer electrolytes for ambient temperature lithium batteries by solvent-free, “one pot” preparation. J. Energy storage,2019, **26**, 100947.
https://doi.org/10.1016/j.est.2019.100947
8. L. Zhang, J. Feng, G. Zhu, J. Yan, S. Bartlett, Z. Wang, Z. Hao, Z. Gao, Effect of $Li_{6.4}La_3Zr_{1.4}Ta_{0.6}O_{12}$ fillers on the interfacial properties between composite PEO-LiTFSI electrolytes with Li metal during cycling. ACS Appl. Mater. Interfaces, 2024, **16**, 13786-13794.
https://doi.org/10.1021/acsami.3c19519
9. L. Liu, D. Zhang, J. Zhao, J. Shen, F. Li, Y. Yang, Z. Liu, W. He, W. Zhao, J. Liu, Synergistic effect of lithium salts with fillers and solvents in composite electrolytes for superior room-temperature solid-state lithium batteries. ACS Appl. Energy Mater., 2022, **5**, 2484-2494.
https://doi.org/10.1021/acsaem.1c04001
